# A Metabolomics Study of Serum in Hospitalized Patients With Chronic Schizophrenia

**DOI:** 10.3389/fpsyt.2021.763547

**Published:** 2021-12-15

**Authors:** Naomichi Okamoto, Atsuko Ikenouchi, Keita Watanabe, Ryohei Igata, Rintaro Fujii, Reiji Yoshimura

**Affiliations:** ^1^Medical Center for Dementia, University Hospital, University of Occupational and Environmental Health, Kitakyushu, Japan; ^2^Department of Psychiatry, University of Occupational and Environmental Health, Kitakyushu, Japan; ^3^Open Innovation Laboratory, Kyoto University, Kyoto, Japan

**Keywords:** schizophrenia, metabolomics, metabolome, glutamate, tetrahydrouridine

## Abstract

**Purpose:** Metabolomics has attracted attention as a new method for understanding the molecular mechanisms of psychiatric disorders. Current metabolomics technology allows us to measure over hundreds of metabolites at a time and is a useful indicator of the consequences of complex and continuous changes in metabolic profiles due to the execution of genomic information and external factors of biological activity. Therefore, metabolomics is imperative to the discovery of biomarkers and mechanisms associated with pathophysiological processes. In this study, we investigated metabolites changes in hospitalized patients with chronic schizophrenia compared to that in healthy controls, and examined the correlations between the metabolites and psychiatric symptoms.

**Patients and Methods:** Thirty patients with schizophrenia and ten healthy controls participated in this study between September 2019 and June 2020. The mean duration of disease in patients with schizophrenia was 26 years. Clinical and neuropsychiatric symptoms of patients with schizophrenia were assessed using the Positive and Negative Syndrome Scale (PANSS). Metabolomics was conducted using Capillary Electrophoresis Fourier Transform Mass Spectrometry (CE-FTMS), using serum samples from patients with schizophrenia and healthy controls. Metabolomics assigned a candidate compound to the 446 (cation 279, anion 167) peaks. Hierarchical cluster analysis (HCA), principal component analysis (PCA), logistic regression analysis, receiver operating characteristic (ROC) analysis, and linear regression analysis were used to analyze the metabolites changes, identifying the disease and the relationship between metabolites and psychiatric symptoms.

**Results:** HCA showed that approximately 60% of metabolites had lower peak values in patients with schizophrenia than in healthy controls. Glutamate metabolism and the urea cycle had the highest proportions in the metabolic pathway, which decreased in patients with schizophrenia. PCA showed a clear separation between patients with schizophrenia and healthy controls in the first principal component (the contribution ratio of the first principal component was 15.9%). Logistic regression analysis suggested that the first principal component was a predictor of disease (odds = 1.36, 95%CI = 1.11–1.67, *p* = 0.0032). ROC analysis showed a sensitivity of 93% and a specificity of 100% for the diagnosis of schizophrenia with a cut-off value of the first principal component; −3.33 (AUC = 0.95). We extracted the high factor loading for the first principal component. Gamma-glutamyl-valine (γ-Glu-Val) was significantly negatively correlated with PANSS total scores (*r* = −0.45, *p* = 0.012) and PANSS general scores (*r* = −0.49, *p* = 0.0055). Gamma-glutamyl-phenylalanine (γ-Glu-Phe) was significantly negatively correlated with PANSS total score (*r* = −0.40, *p* = 0.031) and PANSS general score (*r* = −0.41, *p* = 0.025). Tetrahydrouridine was significantly positively correlated with PANSS negative scores (*r* = 0.53, *p* = 0.0061).

**Conclusion:** Metabolites changes in hospitalized patients with chronic schizophrenia showed extensive and generalized declines. Glutamate metabolism and the urea cycle had the highest proportions in the metabolic pathway, which decreased in the schizophrenia group. Metabolomic analysis was useful to identify chronic schizophrenia. Some glutamate compound metabolites had a relationship with psychiatric symptoms.

## Introduction

Schizophrenia is a severe psychiatric disorder that has a profound impact on individuals and the society (1), however the pathophysiological processes is still unknown.

The complete set of low molecular weight metabolites present in cells and body fluids is called the metabolome, which is usually low molecular weight metabolites of < 1,000 DA in biological samples, such as amino acids, amines, nucleic acids, and sugars. These small molecular chemical entities transcend the genome and proteome, representing the most downstream stage in this dynamic system, defined as metabolism (2). Metabolomics provides an instantaneous snapshot of the physiological status of an organism at a certain time (3). Since metabolomics is direct chemical expression of altered nutritional status and disrupted homeostasis in the body, they have certain advantages over other analytical methods. Changes in metabolic profiles due to the execution of genomic information, many small molecules are directly absorbed by the body, and therefore have functionality in their own right. Furthermore, several diseases are caused by abnormal metabolites levels. Current metabolomics technology allows us to measure over hundreds of metabolites at a time (4) and is a useful indicator of the consequences of complex and continuous changes and external factors of biological activity. Therefore, metabolomics is at the forefront of the discovery of biomarkers and mechanisms associated with pathophysiological processes (5).

Several studies have been performed to identify metabolites changes and biomarkers in schizophrenia (6, 7). Abnormal biological levels have been reported in patients with schizophrenia for several metabolites, including N-acetyl aspartate, lactic acid, tryptophan, glutamic acid, phospholipids, kynurenine, and creatine (8–10). However, despite the large number of reports, there is still a lack of consistent views examining metabolites changes in schizophrenia, potential biomarker metabolites, and their relationship to specific psychiatric symptoms. In addition, to the best of our knowledge, no metabolomics study exists on hospitalized patients with chronic schizophrenia.

This study investigated metabolites changes in hospitalized patients with chronic schizophrenia compared to healthy controls, and examines the correlations between the metabolites and psychiatric symptoms in the patients.

## Materials and Methods

### Ethics Statement

This study was conducted in accordance with the Declaration of Helsinki, and the protocol was approved by the Ethics Committee of the University of Occupational and Environmental Health, Kitakyushu, Japan (UOEHCRB19-024).

### Participants

Thirty patients with schizophrenia recruited from the Komine-Eto and Shin-Moji hospitals participated in this study. All patients were diagnosed with schizophrenia using the Diagnostic and Statistical Manual for Mental Disorders, Fifth Edition (DSM-5). Exclusion criteria included a history of major neurological disease, uncontrolled major medical illness, epilepsy, cerebrovascular accident, head trauma with cognitive sequelae, and mental retardation. All the participants were taking antipsychotic medications. Ten healthy controls, who did not currently have a DSM-5 applicable psychiatric diagnosis or a family history of psychiatric disease participated in the study. Age and sex of the controls were matched to patients as closely as possible, however no special consideration was given to previous physical diseases. None of the patients with schizophrenia and healthy controls had a current smoking habit.

### Clinical Assessment and Blood Sampling

Clinical and neuropsychiatric symptoms of patients with schizophrenia were assessed using the Positive and Negative Syndrome Scale (PANSS) and the Drug Induced Extra-Pyramidal Symptoms Scale (DIEPSS) (11, 12). All blood samples were taken between 9 and 11 a.m., after breakfast and before lunch. The participants fasted and rested for at least 30 min before blood collection. The patient's blood was collected by plain blood tube and serum samples were separated by centrifugation at 2,000 × g for 20 min. Separated serum samples were stored frozen at −80°C in silicone-coated tubes until analysis.

### Measurement of Metabolites Analysis of Blood Samples

The samples obtained at the University of Occupational and Environmental Health were transferred to Human Metabolome Technologies Inc. (HMT; Tsuruoka, Japan). Metabolites analysis was conducted according to the HMT's ω Scan package, using Capillary Electrophoresis Fourier Transform Mass Spectrometry (CE-FTMS) based on the methods (13). Capillary Electrophoresis Fourier Transform Mass Spectrometry analysis was carried out using an Agilent 7100 CE capillary electrophoresis system equipped with a Q Exactive Plus (Thermo Fisher Scientific Inc., Waltham, MA, USA), Agilent 1260 isocratic HPLC pump, Agilent G1603A CE-MS adapter kit, and Agilent G1607A CE-ESI-MS sprayer kit (Agilent Technologies, Inc., Santa Clara, CA, USA). The systems were controlled using Agilent MassHunter workstation software LC/MS data acquisition for 6200 series TOF/6500 series Q-TOF version B. 08.00 (Agilent Technologies) and Xcalibur (Thermo Fisher Scientific) and connected by a fused silica capillary (50 μm i.d. × 80 cm total length) with commercial electrophoresis buffer (H3301-1001 and I3302-1023 for cation and anion analyses, respectively, HMT) as the electrolyte. The spectrometer was scanned from m/z 50 to 1,000. Peaks were extracted using MasterHands automatic integration software (Keio University, Tsuruoka, Yamagata, Japan) to obtain peak information including m/z, peak area, and migration time (MT) (14). Signal peaks corresponding to isotopomers, adductions, and other productions of known metabolites were excluded, and the remaining peaks were annotated according to the HMT metabolome database based on their *m/z*-values and MTs. The areas of the annotated peaks were then normalized by sensitivity correction of analyzers, sample volumes, tissue weight and the number of cells to obtain relative levels of each metabolite. The detected metabolites were plotted on metabolic pathway maps using VANTED software (15). Metabolomics analysis assigned a candidate compound to the 446 (cation 279, anion 167) peaks.

For 168 of the 446 substances, the detected peaks were classified into the following metabolic pathway based on the candidate compounds: carbohydrate metabolism, glucose metabolism/gluconeogenesis metabolism, energy storage by TCA cycle, energy conversion by TCA cycle, glutamate metabolism and urea cycle, choline metabolism and methionine cycle, aromatic amino acids (tryptophan metabolism), aromatic amino acids (phenylalanine and tyrosine metabolism), nucleic acid metabolism (purine metabolism—adenosine), nucleic acid metabolism (purine metabolism—guanosine), nucleic acid metabolism (pyrimidine metabolism), and coenzyme metabolism. The classification of metabolic pathway and substance properties was mainly based on the Kyoto Encyclopedia of Genes and Genomes (KEGG) Pathway database (16).

### Data Analysis

We performed statistical analyses using the proprietary MATLAB and R programs (17) and EZR (18). Welch's *t*-test, hierarchical cluster analysis (HCA), and principal component analysis (PCA) were performed using MATLAB and R programs. Other analyses were performed using EZR.

(a) We used Welch's *t*-test and Fisher's exact test for significant differences between the two groups, including participant characteristics. (b) HCA was used to classify similar factors according to the distance between peaks using the unweighted pair group method with arithmetic mean (UPGMA). The measured values (relative area) were standardized, and peaks with similar relative patterns between samples were classified and represented in a heat map. This heat map was set up, with red being the highest measured value and green being the lowest measured value. (c) We performed PCA and created two-dimensional plots for the first and second principal components. Logistic regression analysis was carried out with the presence of schizophrenia as the objective variable and the first principal component as the explanatory variable. Receiver operating characteristic (ROC) analysis was performed using the first principal component. For the first principal component, we searched for the top ten metabolites (20 metabolites in total) based on the absolute values of the positive and negative factor loading. Factor loading means similar to that of the correlation coefficient with the principal component. (d) For the above 20 substances, we examined the correlation with the PANSS-Total (PANSS-T), PANSS-Positive (PANSS-P), PANSS-Negative (PANSS-N), and PANSS-General (PANSS-G) by Spearman's rank correlation coefficient. The correlation coefficients of these relationships were represented in a heat map in the correlation table. Positive correlations were shown in red, negative correlations were shown in green, and the strength of the correlation was expressed in terms of concentration.

Dates were expressed as the mean (standard deviation). The test was two-tailed, and *p*-value < 0.05 was considered statistically significant. We used Benjamini–Hochberg procedure (19) to control multiple comparisons and expressed the corrected *p*-value as *q*-value. The statistical significance of *q*-value, as well as the *p*-value, was set < 0.05. No outlier was excluded from the analysis. For missing values from the detection limit, only data other than missing values were used in the analysis.

## Results

### Background and Clinical Characteristics

The background and clinical characteristics of the patients with schizophrenia and healthy control groups were shown in Table 1. Disease period was from 10 (minimum) to 43 (maximum) years. No significant differences were observed in background characteristics between the groups.

**Table 1:** Background and clinical characteristics.

	**HC**	**SC**	***p*-values**
	**(*n* = 10)**	**(*n* = 30)**	
Age (years)	48 (8.7)	48 (9.0)	0.99
Sex (males, %)	5 (50%)	17 (57%)	0.73
BMI (kg/m^2^)	23 (2.8)	23 (3.8)	0.89
PANSS			
PANSS-T	–	95 (13)	–
PANSS-P	–	21 (4.6)	–
PANSS-N	–	26 (4.9)	–
PANSS-G	–	48 (8.4)	–
DIEPSS	–	6.3 (3.8)	–
CP total	–	745 (460)	–
Disease period (years)	–	26 (10)	–
Antipsychotic drugs (cases)	–	Risperidone (6) Olanzapine (6) Levomepromazine (5) Clozapine (5) Aripiprazole (5) Haloperidol (4) Quetiapine (3) Zotepine (3) Brexpiprazole (3) Blonanserin (2) Asenapine (2) Fluphenazine (1)	–

*HC, healthy controls; SC, patients with schizophrenia; BMI, body mass index; PANSS, Positive and Negative Syndrome Scale; DIEPSS, Drug Induced Extra-Pyramidal Symptoms Scale; CP, chlorpromazine; Dates were expressed as mean (standard deviation)*.

### Hierarchical Cluster Analysis

**Figure 1:**
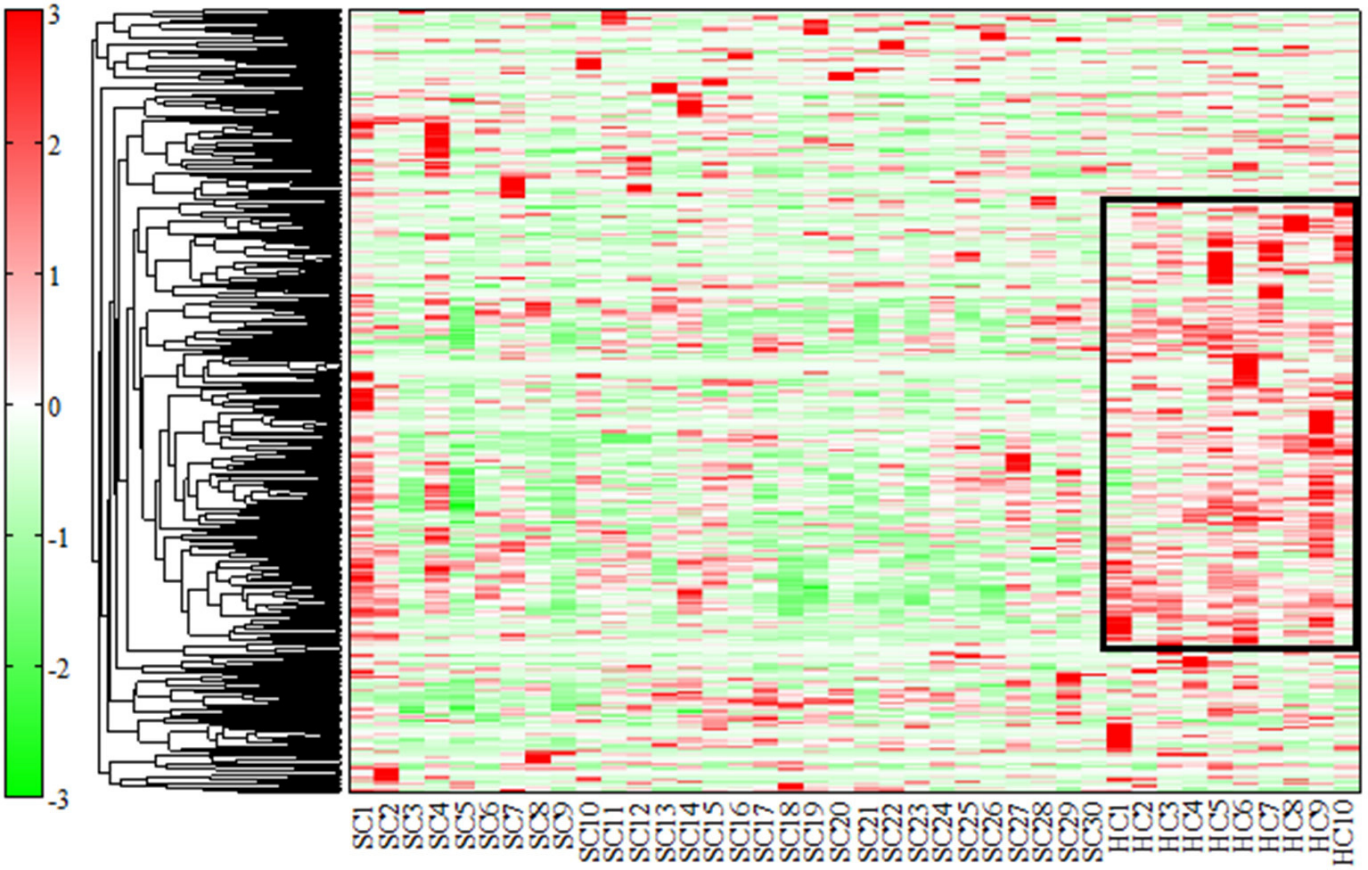
Dendrogram and heat map by hierarchical cluster analysis (HCA). We used HCA to classify similar factors according to the distance between peaks using the unweighted pair group method with arithmetic mean (UPGMA). The measured values (relative area) were standardized, and peaks with similar relative patterns between samples were classified and represented in a heat map. Of the 446 substances measured in the study, 255 were included in the regions with different peak values (black corner). The heat map was set up, with red being the highest measured value and green being the lowest measured value. Most of them were measured at lower levels in patients with schizophrenia than in healthy controls.

**Figure 2:**
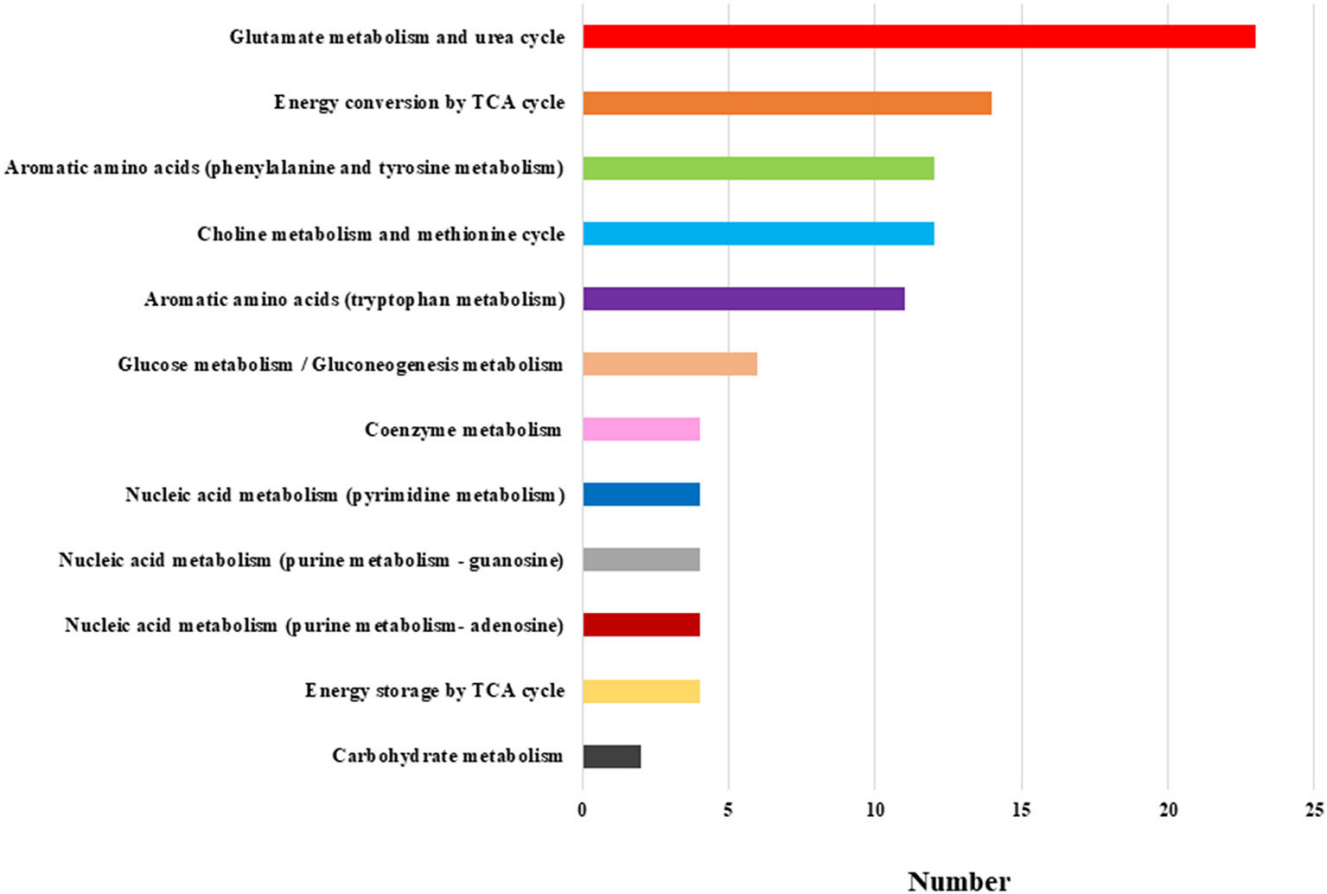
Breakdown of the metabolic pathway of the 82 metabolites. The most frequent breakdown was glutamate metabolism and urea cycle. The next step was energy conversion by the TCA cycle, followed by aromatic amino acids (phenylalanine and tyrosine metabolism), and choline metabolism and methionine cycle.

We evaluated all metabolites using HCA. The dendrogram and heat map were shown in Figure 1. Of the 446 metabolites measured in the study, 255 metabolites were included in the regions with different dendrogram and heat map peak values. Most of them were measured at lower mean levels in the schizophrenia group than in the healthy control group (black corner in Figure 1) [HC, 0.57 (0.32); SC, −0.19 (0.33); *p* = 0.000010; Cohen's *d* = 2.3]. In the frame of 255 metabolites, 82 metabolites were mapped to metabolic pathway. The breakdown of the 82 metabolites was shown in Figure 2. The results showed that glutamate metabolism and the urea cycle had the highest proportions, including the case where metabolites had several pathways.

### Principal Component Analysis

**Figure 3:**
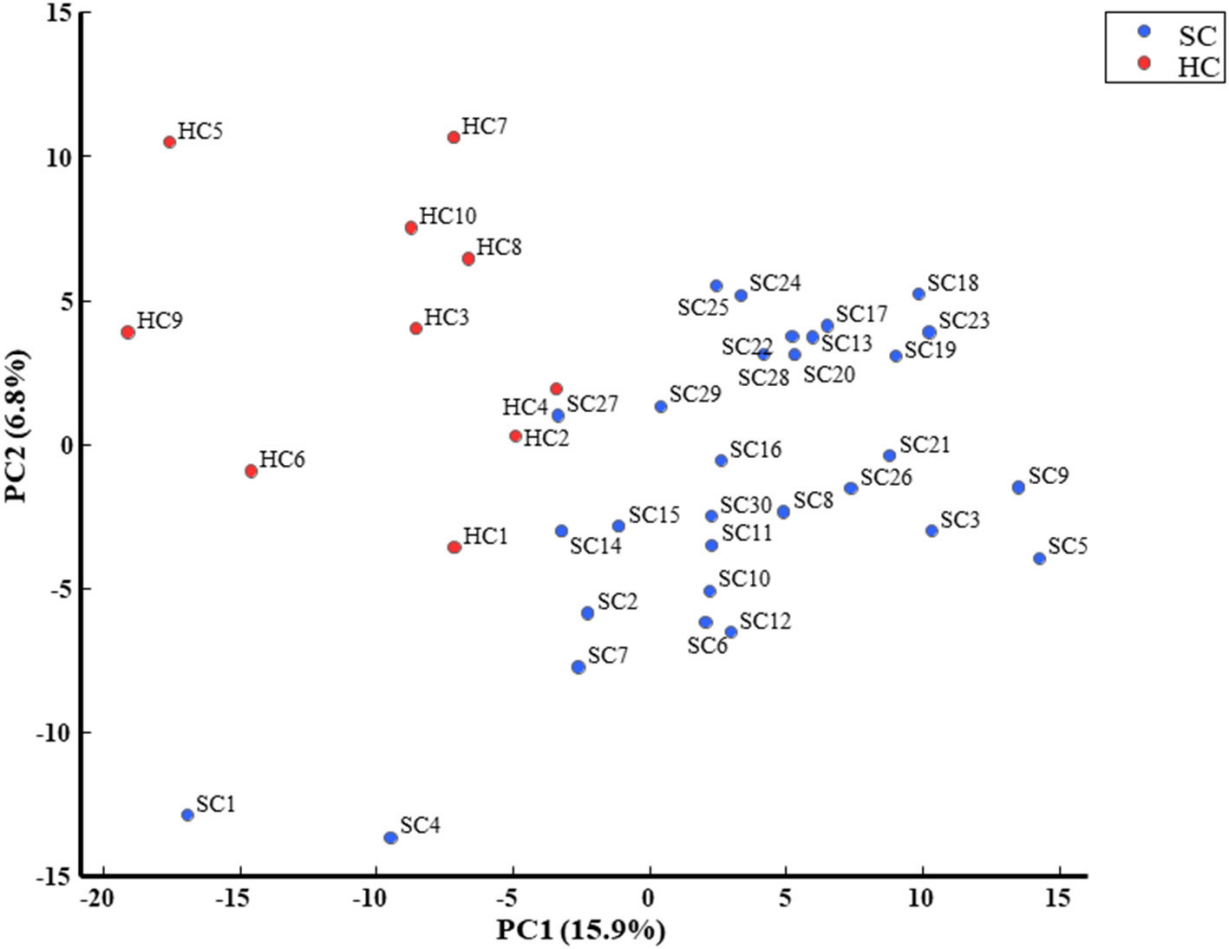
PCA is a multi-dimensional statistical analysis method for unsupervised pattern recognition, which is helpful to understand the total metabolites changing and the variation degree between samples within the group. PCA showed a clear separation between patients with schizophrenia and healthy controls in the first principal component.

**Figure 4:**
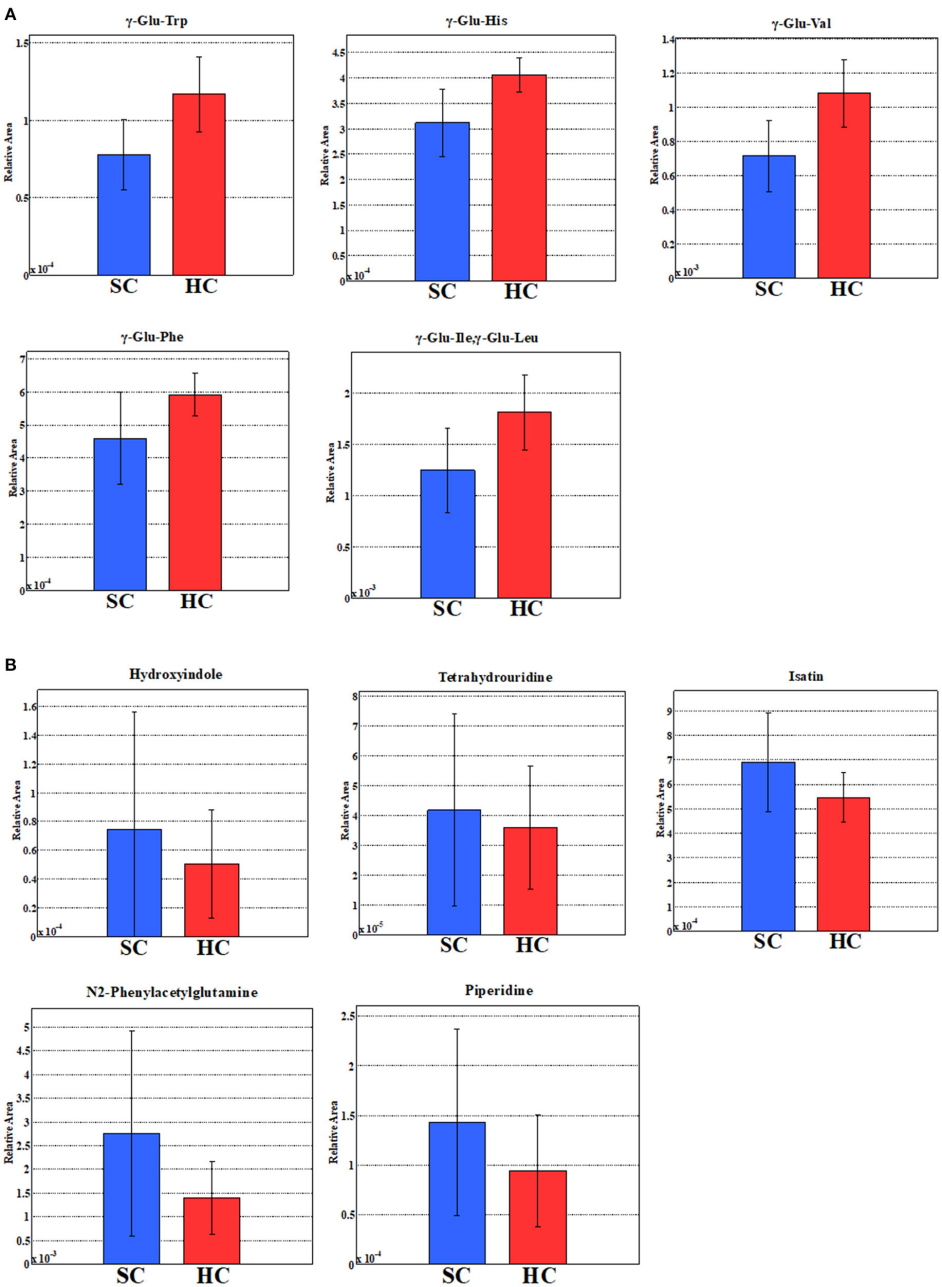
**(A)** Five metabolites with the highest negative factor loading of the first principal component. **(B)** Five metabolites with the highest positive factor loading of the first principal component. The relative areas of the annotated peaks were normalized by sensitivity correction of analyzers, sample volumes, tissue weight, and the number of cells in order to obtain relative levels of each metabolite.

We evaluated all metabolites using PCA and created two-dimensional plots for the first and second principal components. Principal component analysis is a multi-dimensional statistical analysis method for unsupervised pattern recognition, which is helpful to understand the total metabolites changing and the variation degree between samples within the group. PCA showed a clear separation between patients in the schizophrenia group and the healthy control group by the first principal component in Figure 3 [HC, −9.8 (5.4); SC, 3.3 (6.5); *p* = 0.0000057; Cohen's *d* = 2.3]. Logistic regression analysis suggested that the first principal component was a predictor of disease (odds = 1.36, 95%CI = 1.11–1.67, *p* = 0.0032). ROC analysis showed a sensitivity of 93% and a specificity of 100% for the diagnosis of schizophrenia at a cut-off value of the first principal component; −3.33 (AUC = 0.95). We showed the top ten metabolites in positive and negative absolute values of the factor loading of the first principal component (20 substances in total) (Tables 2A,B), and the top five (ten substances in total) metabolites were shown in Figure 4. High negative factor loading metabolites in Figure 4A; higher in the healthy control group than in the schizophrenia group. High positive factor loading metabolites in Figure 4B; higher in the schizophrenia group than in the healthy control group. Factor loading means similar to that of the correlation coefficient with the principal component. The absolute values of the top factor loading of metabolites were greater for negative factor loading than for positive factor loading; The negative factor loading was below −0.70 for all of the top ten substances, while the positive factor loading was as low as 0.38 at maximum. This meant that the negative factor loading was more characteristic of chronic schizophrenia, even with the same top ten. The contribution ratio of the first principal component was 15.9%, and that of the second principal component was 6.8%. Other metabolites with factor loading below −0.70 were isocitric acid (factor loading = −0.71) and glycerol 3-phosphate (factor loading = −0.70).

**TABLE 2A:** Top ten negative factor loading for the first principal component.

**Name**	**Factor loading**	**Pathway**	***HC***	***SC***	***p*-values**	***q*-values**	**Cohen's *d***	**Number of detections**
γ-Glu-Trp	−0.83	–	1.2 × 10^−4^ (2.4 × 10^−5^)	7.8 × 10^−5^ (2.3 × 10^−5^)	0.00046^*^	0.017^*^	1.8	40
γ-Glu-His	−0.82	–	4.1 × 10^−4^ (3.4 × 10^−5^)	3.1 × 10^−4^ (6.7 × 10^−5^)	0.0000024^*^	0.00090^*^	1.6	40
γ-Glu-Val	−0.80	–	1.1 × 10^−3^ (2.0 × 10^−4^)	7.1 × 10^−4^ (2.1 × 10^−4^)	0.00013^*^	0.0096^*^	1.9	40
γ-Glu-Phe	−0.80	–	5.9 × 10^−4^ (6.4 × 10^−5^)	4.6 × 10^−4^ (1.4 × 10^−4^)	0.00029^*^	0.014^*^	1.0	40
γ-Glu-Ile, γ-Glu-Leu	−0.80	–	1.8 × 10^−3^ (3.7 × 10^−4^)	1.2 × 10^−3^ (4.1 × 10^−4^)	0.00074^*^	0.016^*^	1.5	40
XA0004	−0.78	–	1.2 × 10^−4^ (3.0 × 10^−5^)	5.7 × 10^−5^ (2.6 × 10^−5^)	0.0050^*^	0.059	2.3	40
Urea	−0.77	Glutamate metabolism and urea cycle	7.5 × 10^−1^ (2.0 × 10^−1^)	4.5 × 10^−1^ (1.2 × 10^−1^)	0.00094^*^	0.017^*^	2.1	40
N-acetylalanine	−0.77	–	4.9 × 10^−4^ (1.0 × 10^−4^)	4.1 × 10^−4^ (9.5 × 10^−5^)	0.050	0.18	0.4	40
Isethionic acid	−0.77	–	1.1 × 10^−3^ (2.2 × 10^−4^)	7.4 × 10^−4^ (2.2 × 10^−4^)	0.00074^*^	0.017^*^	1.6	40
Creatinine	−0.77	Glutamate metabolism and urea cycle	0.24 (3.6 × 10^−2^)	0.20 (4.7 × 10^−4^)	0.019^*^	0.12	2.3	40

*Factor loading, factor loading means similar to that of the correlation coefficient with the principal component; HC, healthy controls; SC, patients with schizophrenia; γ-Glu-Trp, gamma-glutamyl-tryptophan; γ-Glu-His, gamma-glutamyl-histidine; γ-Glu-Val, gamma-glutamyl-valine; γ-Glu-Phe, gamma-glutamyl-phenylalanine; γ-Glu-Ile, gamma-glutamyl-isoleucine; γ-Glu-Leu, gamma-glutamyl-leucine; XA0004, unidentified peaks that were derived from metabolites but for which no candidate compounds were obtained; γ-Glu-Ile, γ-Glu-Leu, the reason for the two substances was insufficient separation and therefore no peak discrimination; Dates were expressed as mean (standard deviation); We used Benjamini-Hochberg procedure to control multiple comparisons and expressed the corrected p-value as q-value; statistically significant differences at p-value and q-value < 0.05 are indicated by an asterisk*.

**TABLE 2B:** Top ten positive factor loading for the first principal component.

**Name**	**Factor loading**	**Pathway**	***HC***	***SC***	***p*-values**	***q*-values**	**Cohen's *d***	**Number of detections**
Hydroxyindole	0.38	–	5.0 × 10^−5^ (3.8 × 10^−5^)	7.4 × 10^−5^ (8.2 × 10^−5^)	0.45	0.63	0.3	18
Tetrahydrouridine	0.28	–	3.6 × 10^−5^ (2.1 × 10^−5^)	4.2 × 10^−5^ (3.2 × 10^−5^)	0.54	0.70	0.2	34
Isatin	0.26	–	5.5 × 10^−4^ (1.0 × 10^−4^)	6.9 × 10^−4^ (2.0 × 10^−4^)	0.023^*^	0.12	0.8	31
N2-Phenylacetylglutamine	0.25	–	1.4 × 10^−3^ (7.6 × 10^−4^)	2.7 × 10^−3^ (2.2 × 10^−3^)	0.0060^*^	0.058	0.7	40
XA0017	0.25	–	N. D	4.4 × 10^−5^ (2.1 × 10^−5^)	–	–	–	9
Piperidine	0.23	–	9.4 × 10^−5^ (5.6 × 10^−5^)	1.4 × 10^−4^ (9.5 × 10^−5^)	0.065	0.21	0.5	37
Glu-Glu	0.23	–	6.2 × 10^−5^ (2.0 × 10^−5^)	9.6 × 10^−5^ (7.0 × 10^−5^)	0.022^*^	0.13	0.6	40
Melamine	0.23	–	1.2 × 10^−4^ (2.7 × 10^−5^)	1.9 × 10^−4^ (8.7 × 10^−5^)	0.00054^*^	0.015^*^	0.9	37
N2-acetylaminoadipic acid	0.23	–	N. D	1.2 × 10^−4^ (1.0 × 10^−4^)	–	–	–	3
Lipoamide	0.22	–	N. D	1.6 × 10^−4^ (9.8 × 10^−5^)	–	–	–	2

*Factor loading, factor loading means similar to that of the correlation coefficient with the principal component; HC, healthy controls; SC, patients with schizophrenia; XA0017, unidentified peaks that were derived from metabolites but for which no candidate compounds were obtained; Glu-Glu, glutamyl-glutamate; N. D, not detectable; Dates were expressed as mean (standard deviation); We used Benjamini-Hochberg procedure to control multiple comparisons and expressed the corrected p-value as q-value; Statistically significant differences at p-value and q-value < 0.05 are indicated by an asterisk*.

### Relationship Between Metabolites and PANSS Scores

**Figure 5:**
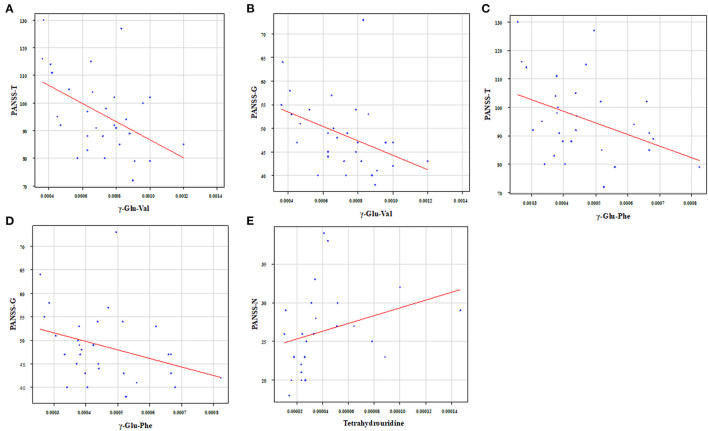
Relationship between metabolites and PANSS. γ-Glu-Val was significantly negatively correlated with **(A)** PANSS-T (*r* = −0.45, *p* = 0.012) and **(B)** PANSS-G (*r* = −0.49, *p* = 0.0055). γ-Glu-Phe was significantly negatively correlated with **(C)** PANSS-T (*r* = −0.40, *p* = 0.031) and **(D)** PANSS-G (*r* = −0.41, *p* = 0.025). Tetrahydrouridine was significantly and positively correlated with **(E)** PANSS-N (*r* = 0.53, *p* = 0.0061).

We showed the relationship between metabolites and PANSS (Tables 3A,B). Gamma-glutamyl-valine (γ-Glu-Val) was significantly negatively correlated with PANSS-T (*r* = −0.45, *p* = 0.012) and PANSS-G (*r* = −0.49, *p* = 0.0055) (Figures 5A,B). Gamma-glutamyl-phenylalanine (γ-Glu-Phe) was significantly negatively correlated with PANSS-T (*r* = −0.40, *p* = 0.031) and PANSS-G (*r* = −0.41, *p* = 0.025) (Figures 5C,D). Tetrahydrouridine was significantly and positively correlated with PANSS-N in Figure 5E (*r* = 0.53, *p* = 0.0061).

**TABLE 3A:** Relationship between metabolites and Positive and Negative Syndrome Scales score.

	**PANSS-T**	**PANSS-P**	**PANSS-N**	**PANSS-G**
γ-Glu-Trp	N. A	N. A	N. A	N. A
γ-Glu-His	−0.10 (*p* = 0.59)	0.22 (*p* = 0.25)	−0.28 (*p* = 0.13)	−0.097 (*p* = 0.61)
γ-Glu-Val	−0.45 (*p* = 0.012)^*^	−0.27 (*p* = 0.15)	−0.27 (*p* = 0.15)	−0.49 (*p* = 0.0055)^*^
γ-Glu-Phe	−0.40 (*p* = 0.031)^*^	−0.27 (*p* = 0.15)	−0.24 (*p* = 0.20)	−0.41 (*p* = 0.025)^*^
γ-Glu-Ile, γ-Glu-Leu	−0.23 (*p* = 0.23)	−0.12 (*p* = 0.54)	−0.12 (*p* = 0.54)	−0.29 (*p* = 0.13)
XA0004	−0.023 (*p* = 0.91)	0.12 (*p* = 0.52)	−0.16 (*p* = 0.39)	0.042 (*p* = 0.83)
Urea	−0.34 (*p* = 0.068)	−0.20 (*p* = 0.29)	−0.25 (*p* = 0.18)	−0.32 (*p* = 0.084)
N-acetylalanine	−0.20 (*p* = 0.29)	0.0058 (*p* = 0.98)	−0.24 (*p* = 0.20)	−0.15 (*p* = 0.43)
Isethionic acid	−0.19 (*p* = 0.33)	−0.081 (*p* = 0.67)	−0.13 (*p* = 0.49)	−0.13 (*p* = 0.50)
Creatinine	−0.28 (*p* = 0.13)	−0.094 (*p* = 0.62)	−0.21 (*p* = 0.26)	−0.35 (*p* = 0.062)

*PANSS, Positive and Negative Syndrome Scale; γ-Glu-Trp, gamma-glutamyl-tryptophan; γ-Glu-His, gamma-glutamyl-histidine; γ-Glu-Val, gamma-glutamyl-valine; γ-Glu-Phe, gamma-glutamyl-phenylalanine; γ-Glu-Ile, gamma-glutamyl-isoleucine; γ-Glu-Leu, gamma-glutamyl-leucine; XA0004, unidentified that were derived from metabolites but for which no candidate compounds were obtained; N.A (Not analyzed), the lines were vertical and no correlation coefficient was calculated; γ-Glu-Ile, γ-Glu-Leu, the reason for the two substances was insufficient separation and therefore no peak discrimination; Positive correlations are shown in red, negative correlations are shown in green, and the strength of the correlation is expressed in terms of concentration; Statistically significant differences at p-value < 0.05 are indicated by an asterisk*.

**TABLE 3B:** Relationship between metabolites and Positive and Negative Syndrome Scales score.

	**PANSS-T**	**PANSS-P**	**PANSS-N**	**PANSS-G**
Hydroxyindole	0.14 (*p* = 0.62)	0.047 (*p* = 0.87)	0.011 (*p* = 0.97)	0.097 (*p* = 0.73)
Tetrahydrouridine	0.33 (*p* = 0.11)	0.044 (*p* = 0.83)	0.53 (*p* = 0.0061)^*^	0.20 (*p* = 0.34)
Isatin	−0.21 (*p* = 0.32)	−0.13 (*p* = 0.55)	−0.25 (*p* = 0.23)	−0.083 (*p* = 0.69)
N2- Phenylacetylglutamine	−0.060 (*p* = 0.75)	0.012 (*p* = 0.95)	−0.24 (*p* = 0.21)	0.10 (*p* = 0.60)
XA0017	0.13 (*p* = 0.74)	0.25 (*p* = 0.52)	−0.31 (*p* = 0.42)	0.075 (*p* = 0.85)
Piperidine	−0.13 (*p* = 0.52)	0.014 (*p* = 0.95)	−0.28 (*p* = 0.16)	0.069 (*p* = 0.73)
Glu-Glu	−0.12 (*p* = 0.54)	−0.13 (*p* = 0.49)	−0.12 (*p* = 0.54)	0.00022 (*p* = 0.99)
Melamine	0.013 (*p* = 0.95)	−0.060 (*p* = 0.76)	0.091 (*p* = 0.64)	0.018 (*p* = 0.93)
N2-acetylaminoadipic acid (*n* = 3)	−0.50 (*p* = 1.0)	0.50 (*p* = 1.0)	−1.0 (*p* = 0.33)	0.50 (*p* = 1.0)
Lipoamide (*n* = 2)	1.0 (*p* = 1.0)	1.0 (*p* = 1.0)	1.0 (*p* = 1.0)	1.0 (*p* = 1.0)

*PANSS, Positive and Negative Syndrome Scale; XA0017, unidentified peaks that were derived from metabolites but for which no candidate compounds were obtained; Glu-Glu, glutalmyl-glutamate; Positive correlations are shown in red, negative correlations are shown in green, and the strength of the correlation is expressed in terms of concentration; Statistically significant differences at p-value < 0.05 are indicated by an asterisk*.

## Discussion

This study investigated metabolites changes in hospitalized patients with chronic schizophrenia compared to that in healthy controls, and examined the correlations between the metabolites and psychiatric symptoms.

The novelty of this study is the examination of serum metabolites changes in long disease period patients with schizophrenia with an average duration of 26 years. HCA showed approximately 60% metabolites with low heat map peak values in the schizophrenia group compared to the healthy control group. These results suggest that the changes in serum metabolites could be one of the pathophysiology of chronic schizophrenia as well as dysfunctions of neuroinflammation, immune systems, or neuroplasticity (20, 21). Glutamate metabolism and the urea cycle had the highest proportions in the metabolic pathway, which decreased in patients with schizophrenia. PCA showed a clear separation between the schizophrenia group and healthy control group in the first principal component. Metabolomic analysis was useful to identify chronic schizophrenia. Among the metabolites with the highest factor loading, γ-Glu-Val, γ-Glu-Phe, and tetrahydrouridine showed statistically significant correlations with PANSS scores. Serum glutamate compound metabolites were inversely correlated with PANSS scores.

The results of the present HCA and PCA suggested that there was an imbalance of diverse metabolites in patients with chronic schizophrenia. Particularly, pathway mapping of metabolites showed that glutamate metabolism and urea cycle were the most abundant, and many of the top substances with high negative factor loading were also glutamate compounds. Alterations in glutamatergic neurotransmission, especially focusing on the *N*-methyl-d-aspartate receptor (NMDAR) function, could be a critical causative feature of schizophrenia (22). Research into the neurobiological processes that could lead to the onset of schizophrenia emphasizes the glutamatergic system and brain development. Clinical research has suggested that altered brain glutamate levels could be present before the onset of psychosis and relate to outcomes in patients at clinical high risk (23). After psychosis onset, glutamate dysfunction could also be related to the degree of antipsychotic response and clinical outcome. These findings support ongoing efforts to develop pharmacological interventions that target the glutamate system and suggest that glutamate compounds could be more effective in specific patient subgroups or disease stages (23). A decrease in plasma glutamate levels was observed in patients with schizophrenia during the first psychotic episode (24). Moreover, plasma glutamate levels were restored after treatment in all instances. Decreased plasma glutamate levels during the first psychotic episodes could reflect impaired glutamate signaling during the initial stages of schizophrenia (24). The results of the present study in patients with chronic schizophrenia found negative correlations between several glutamate compound metabolites in serum and the PANSS scores were compatible with the previous report in the first episode of schizophrenia. In addition, tetrahydrouridine was positively correlated with the PANSS-N scores. Tetrahydroxyuridine, a reversible inhibitor of cytidine deaminase, could exacerbate the negative symptoms of schizophrenia. Tetrahydroxyuridine is known to increase the effect of gemcitabine in tumors, such as pancreatic cancer, when used in combination (25); however, there are no reports on neuronal proliferation. The effects of tetrahydroxyuridine on neurons and glia must be elucidated.

This study has several limitations. The number of participants was only 40, the patients were taking antipsychotic drugs for a long time, and none of the patients with schizophrenia and the healthy controls had a current smoking habit, but not knowing about previous smoking habit, physical diseases, and medication for both groups. The sorting of the metabolites peak values in the HCA was performed by dendrogram and heat map peak. The pattern of metabolites in the data was diverse, including unknown peaks, which could indicate a diverted metabolites in diet or exercise habits. It is necessary at least eight hours fasting before blood collection to avoid the influence of diet. The diverse pattern of metabolites in the data is suggested by the low level of the first principal component of PCA (15.9%). The two patients with schizophrenia had very different distributions in the PCA (SC1 and SC4); however, this could be due to an initial diagnostic error. Moreover, the classification of metabolic pathway and metabolites were based on the KEGG PATHWAY Database (16) and do not take into account currently undiscovered and uncategorized. We must perform further study considering above points to reconfirm the present preliminary results.

In conclusion, the serum metabolites of hospitalized patients with chronic schizophrenia had a diverse decline compared to that in healthy controls. Glutamate metabolism and the urea cycle had the highest proportions in the metabolic pathway, which decreased in the schizophrenia group. Metabolomic analysis was useful to identify chronic schizophrenia. Negative correlations were observed between several glutamate compound metabolites in the serum and psychiatric symptoms in schizophrenia patients.

## Data Availability Statement

The original contributions presented in the study are included in the article/supplementary material, further inquiries can be directed to the corresponding author.

## Ethics Statement

The studies involving human participants were reviewed and approved by the Ethics Committee of the University of Occupational and Environmental Health, Kitakyushu, Japan (UOEHCRB19-024). The patients/participants provided their written informed consent to participate in this study.

## Author Contributions

NO and RY: conceptualization. NO: methodology, software, and visualization. NO, AI, and RY: validation and writing—original draft preparation. NO, RI, and RF: data curation. AI, KW, and RY: writing—review, editing, and supervision. All authors have read and agreed to the published version of the manuscript.

## Conflict of Interest

The authors declare that the research was conducted in the absence of any commercial or financial relationships that could be construed as a potential conflict of interest.

## Publisher's Note

All claims expressed in this article are solely those of the authors and do not necessarily represent those of their affiliated organizations, or those of the publisher, the editors and the reviewers. Any product that may be evaluated in this article, or claim that may be made by its manufacturer, is not guaranteed or endorsed by the publisher.
